# The Book World of Medicine and Science

**Published:** 1900-01-27

**Authors:** 


### The Book World of Medicine and Science.

A Manual of Modern Gastric Methods. By A. Lockhart Gillespie, M.D. (Edinburgh: Oliver and Boyd. 1899. Pp. 175. Illustrated. Price 5s.)

This little manual is not intended as a treatise on diseases of the stomach, or their diagnosis or cure. It bears to diseases of the stomach the relation that a work on urine-testing does to diseases of the kidney—it is a manual of technique. That being so, we perhaps can hardly grumble at the fact that Dr. Lockhart Gillespie rather evades any attempt at justification of the wondrous manœuvres executed by many "stomach specialists" on their suffering patients. What he does do is to give in small compass an admirably lucid and accurate account of these various manœuvres, so that those who have discernment may employ them at the proper time. A perusal of this little volume almost startles us by showing how far specialists have travelled since the pioneer days of Ewald and others. We read of new instruments and methods, of gyromeles and "motor meters," of resuscitators and vibrators; yet, though it is far from our mind to wish to decry accurate diagnosis, we confess to a lurking preference for alkalies and bitters over wonderful wheels and pumps driven by electricity. And it is, we fancy, from the diagnostician rather than the therapeutist that justification of "modern gastric methods" must come. However, we know of no better guide to these methods, diagnostic and quasi-therapeutic, than this little work of Dr. Lockhart Gillespie's. An admirable chapter is contributed (on the mechanical methods used in young children) by Dr. Thomson, and our only complaint against the author is that he has unduly repressed his critical functions in his zeal for cyclopædic completeness.

A Dictionary of Medical Terms. By the late R. D. Hoblyn, M.A. Thirteenth edition. Revised by J. Price, M.D. Pp. 838. Price 10s. 6d. (London: Whittaker and Co. 1899.)

With pleasure we welcome the thirteenth edition of "Hoblyn's Medical Terms," and cordially recognise that in the hands of a new and worthy editor the scholarship of its learned author has suffered no obscuration. Many additions have been made by Dr. Price, particularly in the domain of bacteriology, and none of the newer definitions are other than such as may be supported by authority. But, while we are loth to quarrel with Dr. Price for respecting the labours of Mr. Hoblyn, we confess to some regret that the advent of a medical editor should not have synchronised with a disappearance of some of the older and less accurate definitions. For, whether "Hoblyn" be intended for the instruction of the curious layman or the convenience of the busy practitioner, scientific accuracy and completeness are no less necessary than learned and sometimes remote etymological subtleties. We pursue at random a few references, and find "Hutchinson's teeth" defined as "the notched central incisors" of congenital syphilis. But "Hutchinson's teeth" may refer to the teeth of mercurializtion, and, at any rate, the "pegged" teeth of syphilis have as much claim as the "notched" ones for recognition.

"Caisson disease" is by no means certainly due to "anæmia of the spinal cord"; codeine deserves other mention than as a "feeble soporific"; "Kussmaul's coma" is not "diabetic coma," but a particular type of that condition. The definitions of delusion and illusion given on the authority of Whately are not satisfactory; in fact, grossly misleading. It is true that Hunter's operation involves ligation at a distance from an aneurism, but it should be stated that the ligation is between the aneurism and heart. We are not sure that Mr. Tweedy would accept the disquisition on hemeræopia, and we are quite sure that, if a layman, that on elephantiasis would leave us hopelessly befogged. We intend these criticisms in no carping spirit, but a work of this nature can only justify its existence by completeness and accuracy. However the editor may excuse the absence of an account of the tumours known as chloromata, we fancy he would be hard put to it to justify the omission of "haematoma," which we discovered in a vain attempt to trace some reference to what is known as the "insane ear." But there are doubtless many years of vitality, and many successive editions in store for "Hoblyn" if the definitions are subjected to strict revision at the hands of the editor in accordance with the exactitude demanded nowadays in the medical schools.

The Hæmorrhages of Pregnancy, Parturition, and the Puerperium: The treatment of puerperal eclampsia by saline infusions, with notes of seventeen cases. By Robert Jardine, M.D., Physician to the Glasgow Maternity Hospital. (Edinburgh: William Clay. 1899. 1s. 6d. net. 8vo., pp. 76. Two plates.)

This volume comprises six clinical lectures given at the Glasgow Maternity Hospital. There is no department which is more usefully taught by means of published "clinical lectures" than gynæcology and midwifery. The teachers in Scotch schools have preserved a high standard both in obstetrical instruction and in formal clinical teaching; the traditions in both particulars are well maintained by Dr. Jardine's lectures, which are full of practical points demanding some special reference. The pages on accidental hæmorrhage are extremely valuable; concealed accidental bleeding from rupture of the placental attachment is a rare occurrence, but one so formidable that it behoves every practitioner to know the utmost on the subject. A proper and early recognition of the condition would save a far larger proportion of the cases if they were dealt with courageously. In dealing with this dangerous condition, as in placenta prævia, Dr. Jardine enforces an excellent principle, that is, after rapid dilatation of the cervix by air-bags and turning, delivery should be slow, and the traction on the fœtal feet should be accompanied by pressure on the uterus from above. To lacerate the cervix is to add a grave additional danger. On reading these lectures we should realise the possible dangers of rupture of dilating air-bags, a danger which in these days of decidedly inferior india-rubber goods may become more manifest. We are glad to find here an unqualified condemnation of Lawson Tait's dictum that total extirpation of the uterus is the best treatment of placenta prævia. It is inconceivable that the death-rate claimed by the Birmingham surgeon still exists in these cases, or that any practitioner less practised than he was could expect such good returns from such an operation.

The chapter on eclampsia is excellent. The gist of it is to emphasise the enormous value of large hypodermic injection of saline solution, and the conspicuous dangers of the use of morphia. In lectures of this class we do not expect a complete exposition of all symptoms and procedures, but the consideration of the early hæmorrhage of pregnancy should have included "endometritis," persistent into pregnancy, which has been misleadingly dubbed endometritis gravidanum. The plates (by Dr. J. Lindsay) are wash drawings photographed, but so good that we supposed at first glance that they were direct photographs of spirit preparations. We hope Dr. Jardine will save up some more of these lectures, and issue them in larger bulk. A better eighteen-pennyworth for practitioners who have to deal with midwifery emergencies might be long waited for.

